# Incidence Rate, Survival Rate, and Predictors for Virological Failure Among Adult TB/HIV Coinfected Clients

**DOI:** 10.1155/jotm/2011556

**Published:** 2025-02-15

**Authors:** Nurye Seid Muhie

**Affiliations:** Department of Statistics, Mekdela Amba University, Tulu Awulia, Ethiopia

**Keywords:** adult patient, coinfected, incidence rate, TB/HIV, virological failure

## Abstract

**Background:** Tuberculosis increases human immunodeficiency virus replication and accelerates human immunodeficiency virus progression in both tuberculosis and human immunodeficiency virus coinfected patients. The objective of this study was to determine the incidence rate, survival rate, and predictors for virological failure among adult tuberculosis/human immunodeficiency virus coinfected clients.

**Methods:** A retrospective cohort study was conducted at the University of Gondar Compressive Specialized Hospital from March 2017 to 2022. Secondary data sources were extracted based on inclusion criteria for adult tuberculosis/human immunodeficiency virus coinfected patients. The Cox proportional hazards model was used for adult tuberculosis/human immunodeficiency virus coinfected patients data.

**Result:** The overall incidence rate of virological failure was 9.23 per 1000 person-months observations. Out of 148 coinfected patients, about 24.3% had virological failure. More than half of the patients, 52.7% and 54.1% in this study had a CD4 cell count ≥ 200/mm^3^ and a weight < 50 kg, respectively. Gender (hazard ratio = 1.3291, 95% CI: 1.1878–1.4873), bedridden functional status (hazard ratio = 4.7174; 95% CI: 1.2263–14.1470), WHO clinical Stage IV (hazard ratio = 1.1122, 95% CI: 1.2072–5.9693), patients with opportunistic infections (hazard ratio = 1.2849, 95% CI: 1.4289–3.8504), cotrimoxazole preventive therapy users (hazard ratio = 0.2039, 95% CI: 0.0496–0.8386), patients disclosure status (hazard ratio = 0.1609, 95% CI: 0.0279–0.9286), baseline viral load count < 1000 (hazard ratio = 0.0819, 95% CI: 0.3619–0.8447), and CD4 cell count ≥ 200 (hazard ratio = 0.2728, 95% CI: 0.0749–0.9924) were significant predictors at 5% level of confidence for time to virological failure.

**Conclusion:** The incidence and survival rate of virological failure were high. The current study revealed that male coinfected patients, bedridden functional status, WHO clinical Stage IV, and opportunistic infections other than tuberculosis were associated with a higher time to virological failure while patients disclosed the disease to a family member, cotrimoxazole preventive therapy users, baseline viral load < 1000 copies/mL, and CD4 cell count ≥ 200/mm^3^ had significantly lower time to virological failure. Therefore, public health organizations should be given special attention based on these important predictors to improve their health and prolong the lives of coinfected patients.

## 1. Background

Next to human immunodeficiency virus (HIV), tuberculosis (TB) is also one of the top 10 infectious disease–related deaths worldwide [1]. In 2020, an estimated 8% of all incident cases of TB worldwide occurred among people living with HIV PLWHIV, where Africa stands the highest. In 2021, Africa had an estimated 42 TB/HIV incidence rates (IR) per 100,000 population [1]. The IR of TB/HIV patients in sub-Saharan Africa and South Africa was 5.4 and 4.0 cases per 100 person-years, respectively, before and after starting combined antiretroviral therapy (ART) [2, 3]. Ethiopia ranks among the high burden TB/HIV coinfections with an estimated incidence of both HIV and TB of 8.6 per 100,000, and 2.2 per 100,000 HIV-related TB deaths in 2020 [1]. Then, in high-burden countries, TB/HIV incidence rises in early-maturity populations [4].

TB increases HIV replication and accelerates HIV progression in both TB and HIV coinfected patients. HIV also increases the risk of recurrent TB due to the increased risk of reinfection [5, 6]. Due to TB/HIV progression, TB/HIV coinfected retains high viral load [7, 8]. Hence, virological failure (VF) is defined as a patient's viral load count greater than or equal to 1000 copies viral RNA/mL or greater than or equal to 5000 copies of viral RNA/mL of blood for dry blood spots [9], indicating that HIV is not under control with the current ART regimen and that TB is a major factor for HIV patients.

The 95-95-95 targets set by UNAIDS also specify that 95% of PLWHIV, including those coinfected with HIV/TB know their HIV status, 95% of those who know their status on ART, and 95% achieve viral load below 1000 copies/mL [10]. Then, according to UNAIDS, 95% viral load below 1000 copies/mL is the suppression of viral load and the remaining 5% is VF. However, these UNAIDS targets are not fully satisfied due to high VF in various regions [11]. For instance, in Ethiopia, there is also a high VF in eastern Shewa and Arba Minch with failure rates of 19.1% and 28%, respectively [12, 13]. Then, VF remains a global public health problem, particularly in sub-Saharan African countries [14].

Various predictors have been related to VF in different studies. Among some of them, predictors were older age, patients with other comorbid conditions (OCCs), substance addiction [15], poor adherence to ART drugs, longer ART duration, lower CD4 count, coinfected with TB [14], underweight, low CD4 count, advanced WHO clinical stages, anemia, bedridden or ambulatory, isoniazid preventive therapy (INH) [2], drug addiction [16], cotrimoxazole prophylaxis therapy (CPT), educational status [17], poor adherence, nevirapine (NVP) [18], rural residence [19], fair adherence [20], male gender, stigma [21], baseline viral load count > 1000 copies/mL, opportunistic infections (OIs) other than TB, body mass index, types of TB [22–27], and types of ART [28].

There are limitations of studies conducted in terms of IR, survival rate, and predictors for VF among TB/HIV coinfected patients. Therefore, the objective of this study was to determine the IR, survival rate, and predictors for VF among adult TB/HIV coinfected clients.

The results of this study will improve TB/HIV management and benefit a nation working toward effective TB/HIV control. Additionally, this could lower the suffering and financial burden associated with the diseases and offer suggestions to the appropriate authorities for the establishment of healthcare infrastructure to diminish problems related to hospital clinical care and diagnosis.

## 2. Methods

### 2.1. Study Area

This study was conducted at the University of Gondar Comprehensive Specialized Hospital.

### 2.2. Study Design

In this study, an institutional-based retrospective study was conducted.

### 2.3. Study Period

This study was conducted between March 2017 and March 2022.

### 2.4. Inclusion Criteria

This study included all adult TB/HIV coinfected patients whose age groups are greater than 15 years, patients who have started treatment within the treatment follow-up study period, and patients who have fully recorded information regarding the VF, and known patients' follow-up status were included.

### 2.5. Data Collection Procedure and Data Quality

Trained data clerks obtained the relevant sociodemographic, behavioral, and clinical variable information from patient charts. The sufficiency of the checklist was assessed before the actual data collection, and unclear items were changed. The final data extraction format is modified as needed to ensure consistency and completeness, and ART/TB data management verifies the entire format.

### 2.6. Study Population

The study population consisted of coinfected adults with TB and HIV.

### 2.7. Study Participants' Sample Selection

**Figure 1:**
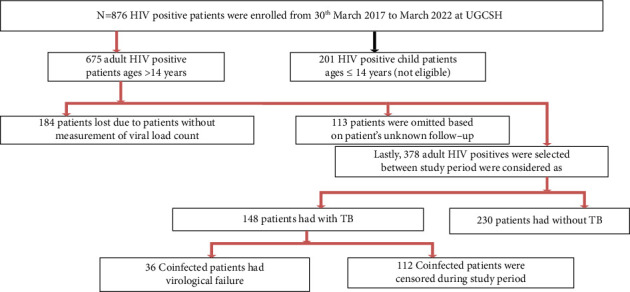
Conceptual framework for adult coinfected patients' sample selection.

Based on the inclusion criteria, 148 study participants were taken into consideration for the current inquiry (Figure 1).

### 2.8. Response Variable

Survival time to VF measured in months.

The survival status of patients can be classified as

(1) $$\begin{array}{c} \text{survival statu}=\left\{\begin{array}{cc} 0, & \text{if censored},\\ 1, & \text{if Virological failure}. \end{array}\right. \end{array}$$

### 2.9. Independent Variables

Gender, age in years, residence, level of education, disclosure, CD4 cell count, baseline viral load count, hemoglobin level, weight, body mass index, WHO clinical stage, treatment adherence, functional status, ART regiment, and OIs other than TB, CPT, INH, TB types, and substance use were considered as independent variables.

### 2.10. Methods of Data Analysis

Data were managed by using SPSS version 28 and imported to R software to analyze the TB/HIV patient's data. Descriptive statistics was used to describe the sociodemographic and clinical characteristics of patients. The Kaplan–Meier method was used to estimate the survival status of VF. The IR of VF was calculated using person months (PM) observation.

### 2.11. Statistical Model

In this study, a semiparametric Cox proportional hazards (PHs) model was used for adult coinfected patients data. Before advancing to another survival statistical model, the GLOBAL test can be used to verify the PH assumption. *p* values greater than 5% suggest that the PH assumption is valid and not rejected by the null hypothesis. Scaled Schoenfeld residuals against the transformed time in month used to show the assumption of Cox PH model satisfied or not.

### 2.12. KM Survival Curve

The KM survival curve graphic indicates that the group defined by the lower curve had a less favorable survival experience than the pattern of the upper curve [29]. It is unlikely to find a difference when the survival curves cross each other.

### 2.13. Univariate Variable Selection

Based on different previous studies [30–32], variables in this study were statistically significant at 25% were chosen for the multivariable analysis after the model was first fitted for each covariate in the univariate variable selection.

## 3. Results

### 3.1. Baseline Clinical and Sociodemographic Characteristics

Out of 148 patients who are coinfected with HIV and TB, 52.7% had a baseline CD4 cell count ≥ 200 cells/mm^3^, of which 9.0% had VF. Similarly, 81.1% had a baseline viral load value of ≥ 1000 copies/mL. Conversely, 26.0% of participants whose hemoglobin level was ≥ 11 g/dL had VF. Approximately, 54.1% and 54.2% of participants had weights ≤ 50 kg and BMIs < 18 kg/m^2^, of which 29.2% and 22.2% had VF during the treatment follow-up period, respectively. Likewise, 32.4% of the study participants were in clinical Stage II, with 20.8% of them having experienced VF. Correspondingly, 38.6% INH and 46.2% CPT drug users were VF (Table 1).

In this study, 64.2% of coinfected patients were female, and 8.4% of them had VF during the treatment follow-up period. Patient's age range between 25 and 34 years were 33.8% and only 12.0% of these individuals had VF. Additionally, 33.1% and 66.9% were residents of rural and urban, respectively. In terms of education level, over 25% had only completed primary school. 74.3% and 39.2% of coinfected patients expressed the status of the disease to family members and nonsubstance users (Table 2).

### 3.2. IR of VF

A total of 3900.0 PM of observations for all the patients of the study followed for different periods. Then, the overall IR of VF in this follow-up study was 9.23 events per 1000 PM of observations. The cumulative hazard of VF at 12, 24, 36, and 48 months were 1.54, 4.62, 2.56, and 0.513 per 1000 PM observations, respectively.

### 3.3. Survival Rate of TB/HIV Coinfected Patients

The rate of VF was 24.3%, and the remainder patients were censored. The VF overall mean and estimated standard errors were 47.416 and 1.723 months, respectively (Table 3).

### 3.4. Kaplan–Meier Estimate of the Survival Function

**Figure 2:**
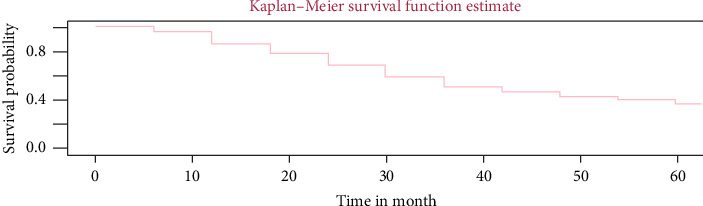
Adult TB/HIV patients' overall survival Kaplan–Meier curve of VF.

In this study, the overall survival Kaplan–Meier curve of VF displayed that the overall survivor function was decreasing monotonically as patient's visit time increases. On the other hand, as the visiting time increases, survival probability of adult HIV positive patients decreased (Figure 2).

### 3.5. Kaplan–Meier Curve for Some Selected Covariates

**Figure 3:**
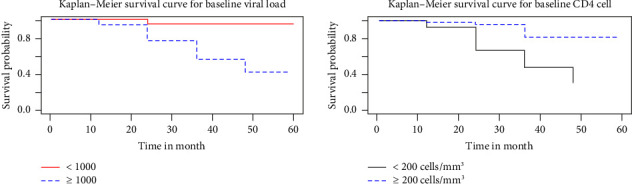
Kaplan–Meier curve for a baseline viral load count and CD4 cell count.

The survival time of patients with viral load < 1000 copies/mL and CD4 cell count ≥ 200 cells/mm^3^ was higher than viral load ≥ 1000 copies/mL and CD4 cell < than 200 cells/mm^3^. On the other hand, in the Kaplan–Meier survival curve below suggested that the above curves are at a lower risk of VF than their counterparts among TBV/HIV coinfected patients (Figure 3).

### 3.6. Evaluations of PHs' Model Assumption

A significant global *p* value greater than 5% (*p* value = 0.42) is obtained from the goodness of the fit test. The proportionality assumption is maintained, according to this global null hypothesis, which is not rejected. Consequently, rather than using other survival models to analyze the survival time to VF, the PH model is more appropriate for this study (Table 4).

**Figure 4:**
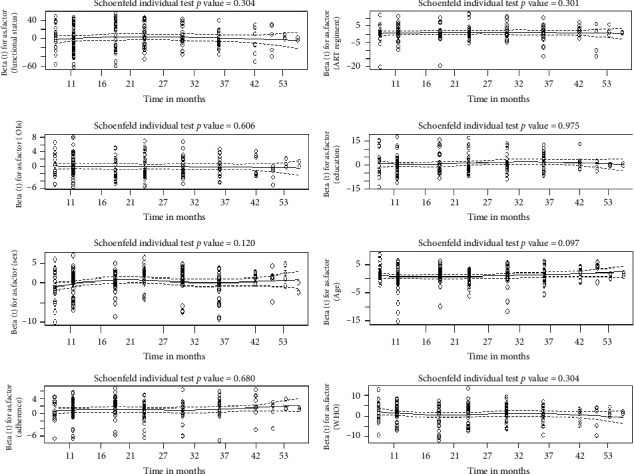
Plots of scaled Schoenfeld residuals against transformed time for some selected covariates.

Scaled Schoenfeld residuals against the transformed time in month for some selected covariates indicated that the solid line is a smoothing-spline fit to the plot, with the broken lines representing a ±2 standard error band around the fit. The plot of Schoenfeld residuals against time for this covariate should not show a pattern of changing residuals for each covariate. Then, the assumption of Cox PH model was satisfied. On the other hand, Schoenfeld residual plots showed that scaled Schoenfeld residuals were randomly distributed and LOESS smoothed curves had no clear departures from the horizontal line recommended that the assumption of PHs appears to be supported for the selected covariates (Figure 4).

### 3.7. Univariable Variable Selection

The covariates gender, age in years, residence, disclosure, baseline viral load count, CD4 cell count, weight, WHO clinical stage, functional status, OIs, substance use, INH, and CPT are statistically significant in the univariable Cox PH model analysis associated with time to VF in TB/HIV coinfected, whereas hemoglobin level, BMI, adherence, ART regimen, TB type, and education were insignificant at a 25% level of significance. These significant univariable covariates were reanalyzed using a multivariable Cox PH model.

### 3.8. Predictors Associated With VF

Gender, functional status, WHO clinical stage, OIs other than TB, CPT, disclosure status, baseline viral load count, and CD4 cell count were significant predictors for time to VF (Table 5). The following interpretations are based on Table 5.

Male coinfected patients had a higher time to VF than female patients, with a hazard ratio of 1.33 (HR = 1.3291, 95% CI: 1.1878–1.4873). On the other hand, patients who expressed their illness to family members had a lower risk of VF as compared to nondisclosure (HR = 0.1609, 95% CI: 0.0279–0.9286).

In comparison to patients with viral load counts ≥ 1000 copies/mL, those with viral load counts < 1000 copies/mL had a lower VF (HR = 0.0819, 95% CI: 0.3619–0.8447). Correspondingly, patients with a CD4 cell count of ≥ 200 cells/mm^3^ were significantly associated with good survival of patients with a hazard ratio of 0.2728 (HR = 0.2728, 95% CI: 0.0749–0.9924).

A higher time to VF was substantially linked with patients in a bedridden functional status (HR: 4.7174; 95% CI: 1.2263–14.1470) as compared to working patients. Similarly, compared to WHO clinical Stage I there was a 1.1 (HR = 1.1122, 95% CI: 1.2072–5.9693) times greater risk of VF in clinical Stage IV coinfected individuals.

Compared to CPT nonusers, there was a 0.2 (HR = 0.2039, 95% CI: 0.0496–0.8386) times lower risk of VF in coinfected individuals who used CPT. When compared to coinfected patients without OIs, patients with OIs had a higher VF (HR = 1.2849, 95% CI: 1.4289–3.8504).

## 4. Discussion

In this retrospective analysis, the time to the VF rate was 24.3%. This indicates that high VF rates in coinfected patients can be concerning. VF typically occurs when the viral load remains above a certain threshold despite TB/HIV treatment. This study is consistent with earlier studies [33]. Then, in this study, predictor's that increase the rate of VF were male TB/HIV coinfected patients, bedridden functional status, WHO clinical Stages-IV, and OIs other than TB.

The overall IR of VF was 9.23 events per 1000 PM observations. The result of this study was around two-fold higher than a study conducted in the Amhara referral Hospital with 4.9 events per 1000 PM observations [18]. Similarly, the IR of this study was higher than the previous study conducted in the St. Paul's Hospital Millennium Medical College, Ethiopia [24], with 1.75 events per 1000 PM observation; in Oromia Region, Ethiopia, with 4.2 events per 1000 PM observation; Addis Ababa, Ethiopia [34], with 3.57 events per 1000 PM observation; in sub-Saharan Africa [2] with 3.49 events per 1000 PM observation; and in the Thailand Chiang Mai University Hospital [35] with 2.33 events per 1000 PM observations. However, the IR of this study was lower than a study conducted in mainland Tanzania [36] with an overall incidence being 16.7 events per 1000 PM observations. These differences might be due to the variation of sample size, study area, and study period. The result of the current study was also more or less comparable with a study conducted in India [37] which reported that the IR of VF was 8.9 events per 1000 PM observations.

In comparison to female patients, male patients experienced a 1.33-fold increase in the time to VF. Male patients who do not consistently take their medication on prescribed time, development of resistance to TB/HIV drugs, lower baseline CD4 cell count, higher baseline viral load, effects of the treatment regimen, and longer duration of ART have been associated with an increased risk of VF. The result of this study is in line with previous studies [38, 39]. This study contradicted earlier research carried out in Ghana [22] and Ethiopia [15]. These contradictions may result from variations in the study regions, study population, and statistical model.

This study also shows that the time to VF is significantly predicted by the baseline viral load count. Patients with low baseline viral load counts (< 1000 copies/mL) had lower VF than those with high baseline viral load counts (> 1000 copies/mL). These results also indicated that coinfected with low baseline viral load counts have larger survival time than high baseline viral load counts and associated with low HIV reservoirs. The results of this study are in line with previous studies [26]. However, the result of this study is inconsistent with previous studies [13].

Results of the current study revealed that baseline CD4 cell count significantly affects the time to VF. The coinfected patients who have CD4 cell count of ≥ 200 cells/mm^3^ had lower VF than those who have < 200 cells/mm^3^, i.e., patients with a better CD4 count are generally associated with better viral suppression and low VF due to patients with better social and clinical condition. These findings agree with the findings of the study conducted in different settings [13, 40, 41].

The present study also found that WHO clinical Stage IV had higher VF compared to clinical Stage I. This might be the case because people with advanced WHO clinical stages have a higher risk of contracting different illnesses and a higher risk of VF. The result of this study is consistent with previous studies [38, 42, 43]. This study also showed that TB/HIV coinfected with OIs are more likely to experience VF than those without OIs. In other expression, patients without OIs had an easier suppression of HIV than patients with OIs. The result of this study is consistent with previous studies [24, 44, 45].

The current study's findings also showed that bedridden coinfected patients had a higher chance of VF than working coinfected patients. Bedridden coinfected patients often face a higher chance of VF compared to those who are working due to weakened CD4 cell count, poor adherence status, advanced disease stage, complications and comorbidities, and nutritional deficiencies. On the other hand, working patients had an appropriate follow-up of ART, and leads to suppressed HIV to undetectable levels of the blood than bedridden patients. This result is in line with previous studies [20, 46–48].

CPT user patients are less likely to experience VF than nonusers. CPT is beneficial for TB/HIV coinfected patients in several ways, which may contribute to a lower likelihood of experiencing VF compared to nonusers due to reduction in OIs, better quality of immune function, improved adherence to ART, and synergistic effects with ART. In other ways, nonusers are associated with a more risk of VF than nonusers. The result of this study is consistent with previous studies [29, 49, 50]. However, this result is contradicted by the previous study [18].

Patients who express their history of the disease to near relatives have less VF than nondisclosing patients. The major reasons why TB/HIV coinfected patients who disclose their disease history to near relatives might experience less VF compared to those who do not disclose were emotional and psychological support, good adherence to treatment, practical aspects of managing the disease, reduce the stigma associated with TB and HIV, and enhancing monitoring. The result of this study is consistent with previous studies [51, 52]. Disclosing one's TB/HIV status to potential sexual partners is crucial for preventing the spread of HIV/TB infection. Acquiring social support from others and promoting compliance and commitment to anti-HIV/TB drugs are also crucial aspects of it. To stop the spread of TB/HIV and to protect the health of those living with TB/HIV, their partners, and the community, it is crucial to disclose one's TB/HIV status. Despite the advantages of disclosure, a large number of HIV-positive individuals put off telling others close to them about their status, which raises the possibility of the virus spreading. This expression is consistent with the previous study [53–57].

## 5. Conclusion and Recommendation

The incidence and survival rates of VF were high. The current study revealed that male TB/HIV coinfected patients, bedridden functional status, WHO clinical Stage IV, and OIs other than TB were associated with higher time to VF while patients who disclosed the disease to a family member, CPT users, viral load < 1000 copies/mL, and those with a CD4 cell count ≥ 200/mm^3^ had significantly lower time to VF. Therefore, public health organizations should be given special attention based on these important predictors to improve their health and prolong the lives of coinfected patients. Finally, this study suggested that health professionals should conduct health-related studies to overcome coinfected patients' suppression of viral load due to prescribed medication and better life of survival.

## 6. Strengths of This Study

This study speaks about the VF IR, survival rate, and predictors among adult TB/HIV coinfected clients. Also, this study can provide better insight and reference by generating local biostatistical, epidemiological, and clinical data for public health policymakers.

## Figures and Tables

**Table 1:** Clinical characteristics of adult coinfected individuals.

Variables	Categories	Survival status		Total (%)
		Censored (%)	Virological failure (%)	
CD4 cell count	< 200	41 (58.6)	29 (41.4)	70 (47.3)
	≥ 200	71 (91.0)	7 (9.0)	78 (52.7)

Baseline viral load	< 1000	27 (94.6)	1 (3.6)	28 (18.9)
	≥ 1000	85 (70.8)	35 (9.0)	120 (81.1)

Hemoglobin level	< 11	75 (76.5)	75 (23.5)	98 (66.2)
	≥ 11	37 (74.0)	13 (26.0)	163 (33.8)

Weight	< 50 kg	56 (70.0)	24 (29.2)	80 (54.1)
	≥ 50 kg	56 (82.4)	12 (17.6)	68 (45.9)

BMI	< 18.5 kg/m^2^	63 (77.8)	18 (22.2)	81 (54.7)
	18.5–24.9 kg/m^2^	28 (66.7)	14 (33.3)	42 (28.4)
	≥ 25	21 (84.0)	4 (16.0)	25 (16.9)

WHO clinical stage	Stage 1	37 (92.5)	3 (7.5)	40 (27.0)
	Stage 2	38 (79.2)	10 (20.8)	48 (32.4)
	Stage 3	29 (61.7)	18 (38.3)	47 (31.8)
	Stage 4	8 (61.5)	5 (38.5)	13 (8.8)

Adherence	Poor	32 (72.7)	12 (27.3)	44 (29.7)
	Fair	49 (75.4)	16 (24.6)	65 (43.9)
	Good	30 (78.9)	8 (21.1)	38 (25.7)

Functional status	Working	77 (90.6)	8 (9.4)	85 (57.4)
	Ambulatory	22 (64.7)	12 (35.3)	34 (23.0)
	Bedridden	13 (44.8)	16 (55.2)	29 (19.6)

ART regiment	1d	35 (77.8)	10 (22.2)	45 (30.4)
	1c	40 (81.6)	9 (18.4)	45 (33.1)
	1e	15 (68.2)	7 (31.8)	117 (14.9)
	Other	22 (68.8)	10 (31.8)	32 (21.6)

INH	No	69 (88.5)	9 (11.5)	78 (52.7)
	Yes	43 (61.4)	27 (38.6)	70 (47.3)

CPT	No	77 (92.8)	6 (7.2)	83 (56.1)
	Yes	35 (53.8)	30 (46.2)	65 (43.9)

OIs other than TB	No	97 (85.8)	16 (14.2)	113 (76.4)
	Yes	15 (42.9)	20 (57.1)	35 (23.6)

TB types	Pulmonary	51 (34.5)	12 (8.1)	63 (42.6)
	Extra-pulmonary	70 (83.3)	14 (16.7)	85 (57.4)

*Note:* 1d refers to ART treatment of AZT-3TC-EFV, 1c refers to ART treatment of AZT-3TC-NVP, 1e refers to TDF-3TC-EFV and other means other ART treatments, like1j (TDF+3 TC + DTG), and 1f (TDF+3 TC + NVP).

**Table 2:** Coinfected patients' baseline sociodemographic characteristics.

Variables	Categories	Survival status		Total (%)
		Censored (%)	Virological failure (%)	
Gender	Female	87 (91.6)	8 (8.4)	95 (64.2)
	Male	25 (47.2)	28 (52.8)	53 (35.8)

Age	15–24 years	10 (45.5)	12 (54.5)	22 (14.9)
	25–34 years	44 (88.0)	6 (12.0)	50 (33.8)
	35–44 years	30 (81.1)	7 (18.9)	37 (25.0)
	> 44 years	28 (71.8)	11 (28.2)	39 (26.4)

Residence	Rural	22 (44.9)	27 (55.1)	49 (33.1)
	Urban	90 (90.9)	9 (9.1)	99 (66.9)

Level of education	Noneducated	24 (52.2)	22 (47.8)	46 (31.1)
	Primary	33 (86.8)	5 (13.2)	38 (25.7)
	Secondary	33 (91.7)	3 (8.3)	36 (24.3)
	Tertiary	22 (78.6)	6 (21.4)	28 (18.9)

Disclosure status	No	11 (22.6)	27 (77.4)	38 (25.7)
	Yes	101 (91.8)	9 (8.2)	110 (74.3)

Substance use	No	84 (93.3)	6 (6.7)	90 (60.8)
	Yes	28 (48.3)	30 (51.7)	58 (39.2)

**Table 3:** Adult TB/HIV coinfected patients' survival status.

Mean in month		Survival status	
Estimate	Std. error	Event (%)	Censored (%)
47.416	1.723	36 (24.3)	112 (75.7)

**Table 4:** Verification of PHs model assumptions.

Variable	Chi-square	DF	*p* value
Age	2.7566	3	0.43
Sex	0.0413	1	0.84
Education	2.7502	3	0.43
Residence	0.5617	1	0.45
Disclosure	0.0802	1	0.78
Baseline viral load	1.4671	1	0.23
Hemoglobin level	0.0107	1	0.92
CD4 cell count	0.0443	1	0.83
Weight	0.3867	1	0.53
BMI	0.8287	2	0.66
Adherence	1.4335	2	0.49
WHO clinical stage	3.2373	3	0.36
ART regiment	1.3117	3	0.73
Functional status	1.5803	2	0.45
Types of TB	0.3196	1	0.57
OIs	0.3126	1	0.58
Substance use	0.3683	1	0.54
GLOBAL	28.883	28	0.42

*Note:* DF indicated the degree of freedom.

**Table 5:** Multivariable Cox PHs' model results for predictors associated with virological failure.

Variables	Categories	Β	S.E	*p* value	HR	95% CI for HR	
						Lower CI	Upper CI
Age (Ref. 15–24)	25–34	−0.98599	0.70240	0.160	0.3731	0.0942	1.4780
	35–44	−0.34288	0.55845	0.709	0.5392	0.2375	2.1205
	> 44	−0.14529	0.50642	0.774	0.8648	0.3205	2.3333

Gender (Ref. female)	Male	0.28451	0.05739	0.040⁣^∗^	1.3291	1.1877	1.4873

Residence (Ref. rural)	Urban	1.16484	0.90762	0.199	3.2054	0.5411	18.987

Disclosure (Ref. No.)	Yes	−1.82702	0.89436	0.041⁣^∗^	0.1609	0.0279	0.9286

Viral load (Ref. ≥ 1000)	< 1000	−2.50217	1.11904	0.025⁣^∗^	0.0819	0.3619	0.8447

CD4 count/mm^3^ (Ref. < 200)	≥ 200	−1.29907	0.65890	0.048⁣^∗^	0.2728	0.0749	0.9924

Weight (Ref. < 50)	≥ 50 kg	0.25900	0.48492	0.593	1.2956	0.5007	3.3516

Functional status (Ref. working)	Ambulatory	0.39345	0.63001	0.532	1.4821	0.4311	5.0949
	Bedridden	1.55127	0.68738	0.024⁣^∗^	4.7174	1.2263	18.147

WHO (Ref. Stage I)	Stage II	−0.14464	0.80718	0.857	0.8653	0.1779	4.2098
	Stage III	0.06936	0.82518	0.933	1.0718	0.2127	5.4015
	Stage IV	0.10630	1.11215	0.004⁣^∗^	1.1122	1.2072	5.9693

INH (Ref. No.)	Yes	−0.83811	0.72023	0.245	0.4325	0.1054	1.7745

CPT (Ref. No.)	Yes	−1.59009	0.72146	0.027⁣^∗^	0.2039	0.0496	0.8386

OIs (Ref. No.)	Yes	0.25068	0.55995	0.014⁣^∗^	1.2849	1.4288	3.8504

Substance use (Ref. No.)	Yes	0.09424	0.74472	0.899	1.0988	0.2114	3.9172

Abbreviations: Ref, reference; S.E, standard error.

⁣^∗^Indicated statistical significance at a 5% level of significance, β indicated coefficient, OIs indicated opportunistic infections other than TB, HR = exp (β) indicated hazard ratio, and CI indicated confidence interval for HR.

## Data Availability

The data that support the fndings of this study are available from the corresponding author upon reasonable request.

## Nomenclature

AIDS: Acquired immune deficiency syndrome
ART: Antiretroviral therapy
BMI: Body mass index
CD4: Cluster differentiation 4
CPT: Cotrimoxazole preventive therapy
HIV: Human immune deficiency virus
IR: Incidence rate
INH: Isoniazid preventive therapy
OIs: Opportunistic infections
PHs: Proportional hazards
PLWHIV: People living with HIV
PM: Person months
SR: Survival rate
TB: Tuberculosis
VF: Virological failure
WHO: World Health Organization
